# Comorbid Bipolar and Alcohol Use Disorder—A Therapeutic Challenge

**DOI:** 10.3389/fpsyt.2021.660432

**Published:** 2021-03-23

**Authors:** Heinz Grunze, Martin Schaefer, Harald Scherk, Christoph Born, Ulrich W. Preuss

**Affiliations:** ^1^Psychiatrie Schwäbisch Hall, Schwäbisch Hall, Germany; ^2^Paracelsus Medical University Nuremberg, Nuremberg, Germany; ^3^Klinik für Psychiatrie, Psychotherapie, Psychosomatik, und Suchtmedizin, Evang. Kliniken Essen-Mitte, Essen, Germany; ^4^Klinik für Psychiatrie und Psychotherapie, Campus Charité Mitte, Charité Universitätsmedizin Berlin, Berlin, Germany; ^5^Vitos Klinikum Riedstadt, Riedstadt, Germany; ^6^Vitos Klinik Psychiatrie und Psychotherapie, Herborn, Germany; ^7^Klinik für Psychiatrie, Psychotherapie, und Psychosomatik, Martin-Luther-Universität Halle-Wittenberg, Halle, Germany

**Keywords:** alcohol use disorder, atypical antipsychotics, bipolar disorder, depression, lithium, psychotherapy, valproate

## Abstract

Comorbidity rates in Bipolar disorder rank highest among major mental disorders, especially comorbid substance use. Besides cannabis, alcohol is the most frequent substance of abuse as it is societally accepted and can be purchased and consumed legally. Estimates for lifetime comorbidity of bipolar disorder and alcohol use disorder are substantial and in the range of 40–70%, both for Bipolar I and II disorder, and with male preponderance. Alcohol use disorder and bipolarity significantly influence each other's severity and prognosis with a more complicated course of both disorders. Modern treatment concepts acknowledge the interplay between these disorders using an integrated therapy approach where both disorders are tackled in the same setting by a multi-professional team. Motivational interviewing, cognitive behavioral and socio- therapies incorporating the family and social environment are cornerstones in psychotherapy whereas the accompanying pharmacological treatment aims to reduce craving and to optimize mood stability. Adding valproate to lithium may reduce alcohol consumption whereas studies with antipsychotics or naltrexone and acamprosate did not affect mood fluctuations or drinking patterns. In summary, there is a continuous need for more research in order to develop evidence-based approaches for integrated treatment of this frequent comorbidity.

## Epidemiology of Bipolar Disorder and Substance Use Disorder

Both bipolar affective disorder (BD) and substance use disorder (SUD) are wide-spread in the general population. Most epidemiological and treatment studies were conducted according to DSM-IV or ICD-10 criteria that distinguishes between substance abuse and dependence as diagnostic entities on its own. Depending on the diagnostic system (ICD or DSM) used and subject sample studied, bipolar affective disorder (BD) in the general population has a lifetime prevalence between 1.3 and 4.5% (1). The World Health Organization World Mental Health Survey Initiative (2) conducted across eleven countries reported a 4.8% lifetime prevalence of all manifestations of bipolarity, including subthreshold and spectrum disorder.

Looking at specific countries, a representative survey applying the Composite International Diagnostic Interview [CIDI (3)] for ICD 10 and DSM-IV criteria reports a 1-year prevalence rate of 1% for BD -I and 0.6% for BD-II disorder for Germany (4). The same study reports on a 1-year prevalence of 5.7% for substance abuse (except nicotine) according to DSM-IV criteria. Three percent fulfilled criteria for alcohol dependence and 1.8% for abuse (4). In a prior survey, looking at lifetime prevalence rate, the same group reports on similar numbers for BD, and 9.9 and 8.5% for alcohol abuse and dependence, respectively (5). These numbers are in a similar range as in other European countries; while prevalence rates from the US are much higher, both for BD and substance abuse/dependence (6). Whereas numbers for legal substances, e.g., alcohol, are considered as relatively robust and reproducible, many cases of illicit drug use remain undetected in patients with BD. Cannabis is likely to be second after alcohol as substance of abuse in BD patients, affecting approximately one quarter of bipolar patients (7).

Whereas, the incidence of BD across countries and cultures is within a similar range, reported rates for AUD differ considerably due to cultural and religious diversity. For example, a representative household survey in Iran found a 12-month prevalence of alcohol use disorders of 1% according to DSM-IV criteria and 1.3% according to DSM-5, with higher prevalence rates in urban vs. rural areas (8). For comparison, a recent US household survey reports a 12-month prevalence of DSM-5 AUD of 13.9% (9).

SUD comorbidity is not exclusive to adult bipolar patients but starts early in life. Pediatric onset BD rarely occurs in the absence of comorbid conditions, and the co-occurrence of additional disorders complicates both the accurate diagnosis of BD and its treatment. Manifestation of BD in children and adolescents is not as infrequent as previously assumed, with rates of bipolar spectrum disorder reaching an estimated 4%, especially in US samples (10).

In the meantime, DSM-5 (11) abolished the distinction between substance use, abuse and dependency by defining threshold numbers of criteria for different grades of severity of substance use. Of the 11 criteria, 2–3 should be fulfilled to diagnose mild alcohol use disorder (AUD) (12). Also, BD criteria experienced some adaptions with yet speculative consequences for epidemiological figures. Whereas, criteria for a manic episode were tightened (13, 14) preceding substance use *per se* is no more an exclusion criterion for a genuine BD diagnosis as long as the mental alterations exceed well the physiological effect of the substance. This may change figures of future epidemiological studies on SUD and BD comorbidity to some degree.

Both disorders follow a chronic course and considerably impair social functioning and quality of life (15–17), general health and ultimately life expectancy (18–20). Antecedent SUD has been associated with earlier age of onset of BD (21) and a greater need of hospitalization at onset of BD (22). In addition, both disorders have a significantly increased rate of suicides and suicide attempts with an added risk in case of coexistence of both disorders (23–25).

## Comorbidity of Bipolar Affective Disorders in Alcohol Use Disorder Patients

Among mental health disorders, BD has probably the highest risk of having a second, comorbid DSM -IV axis I disorder (26). Epidemiological data from the US report life-time prevalence rates of up to 90% for comorbidities in BD (6), with 62.3% for AUD (39.1% for DSM-IV alcohol abuse and 23.2% for alcohol dependency) followed by cannabis (46%), cocaine (24%) and opioids (8.5%) (27). The already cited WHO census across 11 countries showed a mean SUD life time comorbidity with BD of 36.6% with a large variation between countries (2). A meta-analysis including nine national surveys conducted between 1990 and 2015 revealed a mean prevalence of 24% for AUD and of 33% for any SUD except nicotine (28). Analyzing SUD and bipolar comorbidity in clinical settings, the same group reports the highest prevalence for AUD (42%) followed by cannabis use (20%) and any other illicit drug use (17%) (21). Cannabis ranking second after AUD has also been confirmed in other studies (7, 27, 29). Similar rates of SUD were also reported in the Systematic Treatment Enhancement Program Bipolar Disorders (STEP BD) study including 3,750 Bipolar I or II patients (30).

Our own study in BD patients recruited from the Stanley Foundation Bipolar Network (SFBN) outpatient clinic with more comprehensive care found life-time SUD in 42% and AUD in 33% of BD patients (31). Secondary analysis of this data examined the gender-specific relationships between AUD and BD. In line with epidemiological catchment area studies (26), absolute numbers of AUD in males with BD were higher (49 vs. 29%), however, the relative risk of suffering from AUD was significantly greater for women with BD [odds ratio (OR) = 7.35] than for men (OR = 2.77) in relation to the general population (32). Among other factors such has higher rates of depression, previous trauma might play a role for the higher OR in female BD patients; additional analysis in a larger sample of SFBN patients revealed that bipolar women with AUD had also a significantly higher rate of post-traumatic stress disorder (PTSD) than those without AUD (33).

The risk of developing comorbid SUD is obviously much higher in BD than in Major Depressive Disorder (MDD) suggesting that depressive mood is not the main driver of comorbidity in BD, but other factors imminent to BD, such as personality, early onset and impaired functionality, might play a decisive role. The OR for developing a SUD has been estimated 1.8 in patients with a lifetime MDD and 6.9 for those with a lifetime BD-I, compared with the general population (34), and prevalence rates for SUD are ~25–50% higher in BD-I than BD-II patients (26, 35). The latter appears to be mainly driven by illicit drugs (OR 7.46 in BD-I and 3.30 in BD-II) (28). For AUD, however, a recent meta-analysis of 22 studies showed no difference between BD-I (OR 3.78) and BD-II (OR 3.81) (28). A recent catchment area study in Northeast England found a 40% lifetime comorbidity between BD II and AUD, surprisingly with little difference between female (38%) and male (43%) subjects (36).

As mentioned, there is a wide variation of prevalence rates for BD-SUD comorbidity across countries (2) with higher rates in the US than in other industrialized countries. Analyzing the SFBN sample of the two German centers revealed a life-time prevalence of 17.8% for AUD only—compared to 33% in the whole SFBN which included four US and three European centers (two in Germany, one in the Netherlands). The transatlantic difference for illicit drug use might be even higher, as SUD other than AUD was only present in 8.5% of the German SFBN sample (37). The higher SUD comorbidity rates in the US might directly relate to the poorer prognosis and higher treatment resistance in the SFBN US compared to the European sample (38).

Less data has been generated for the rate of BD in samples of AUD patients. Not only that BD pre-disposes for SUD, also the opposite is true: According to the already cited meta-analysis by Hunt and colleagues, people with an AUD were 4.1 times on greater risk of having a BD compared to those without an AUD (21). The US- National Comorbidity Survey (39) found that 6.5% of males and 10.6% of females with alcohol dependency had also suffered from at least one manic episode. As a limitation, this survey did not differentiate between manic episodes which preceded SUD, those which followed SUD and those which were possibly induced by substance use. In addition, it is fair to assume that there is a substantial dark figure as symptoms of BD are often masked by SUD. Recognizing an underlying BD in SUD patients, however, is essential to tailor adequate treatment plans.

## Is There a Shared Etiology Between BD and Aud?

Family studies indicate that AUD and affective disorders, especially BD have a shared genetic pre-disposition. Examining the family history of bipolar patients participating in the SFBN we observed that, among others, AUD not only in parents (40), but also grandparents of a bipolar patient is associated with a more severe course of BD and poor prognosis (41). Temperament traits, prevalent in BD and AUD and genetically determined such as sensation seeking behavior may play a decisive role across illness boundaries (42, 43). Candidate genes include a shared polymorphism of the aldehyde hydrogenase and alcohol dehydrogenase (44), a Ser23Cys (rs6318) polymorphism of the 5HT2C gene (in female bipolar patients) (45) and a VAL-158-MET polymorphism of the cathechol-o-methyltransferase (COMT) (46) impacting on the monoamine metabolism. However, recent GWAS finding pointed out that the correlation between genetic alterations and psychiatric disorders is not simple; the genetic connection of substance use and psychiatric disorders is rather highly pleiotropic and involves shared neurodevelopmental path, neurotransmission, and intracellular trafficking (47).

## Diagnosis of AUD in Bipolar Patients

Symptoms of AUD and SUD may often obscure an underlying diagnosis of BD, and frequently result in a long delay before a BD diagnosis has been established by careful clinical observation. Brown et al. reported rates of SUDs in patients with BD ranging from 14 to 65% in treatment settings (48) but only a minority has received a correct diagnosis so far. Given the high incidence of psychiatric comorbidities in AUD, the German S3 Guideline recommend in every patient with AUD to carefully screen for psychiatric comorbidities after completing treatment of acute intoxication or withdrawal (49).

Uncovering AUD in people with BD appears less problematic. A recent systematic review on comorbidity of BD and AUD (50) recommends the use of the Alcohol use disorder Identification Test [AUDIT, (51, 52)] to detect heavy and frequent alcohol use, and AUDIT appears to be also sensitive in patients with comorbid mental health disorders. The AUDIT is also recommended to screen comorbid individuals by several evidence- based guidelines, e.g., the German S3-Guidelines on AUD (49, 53).

## Consequences of Comorbidity

The detrimental impact of substance use and BD has been well-established, both for the individual and for society (54, 55). Numerous investigations demonstrated that comorbid AUD influences the clinical course of BDs unfavorably [for a review, see (50)]. Especially in younger people BD as well as SUD results in severe and lasting impairment and a loss of healthy years lived (56, 57). BD and SUD are afflicted with high rates of suicide attempts and suicide that are even topped in case of coexistence of both disorders (24). A Brazilian study reports of at least one suicide attempt in 68% of BD patients with AUD compared to 35% in BD without AUD, with virtually no difference between BD patients with DSM-IV alcohol abuse and dependence (23).

Gender differences have a significant influence on treatment outcomes in BD (58) but not as much on outcomes in alcohol dependence (59). AUD also facilitates additional comorbidities in BD patients such as anxiety disorders in female patients (33) and has a detrimental effect on the course of BD in general with an earlier onset (28), delayed recovery from episodes, more frequent mood switches, rapid cycling, mixed states, more severe depression and suicidal ideation (30, 32, 60) and lower adherence to treatment (61). Especially a history of verbal abuse and rates of social phobia and depression are higher in female than male BD patients with AUD (32). Whereas, AUD in female BD patients fosters rather self-destructive consequences, males appear more likely to externalize anger and impulsivity, and stand out by a history of criminal actions (62). Specific numbers for AUD and BD are not available, but for affective disorders (AD) in general and SUD, criminal behavior has been observed twice as frequent in AD with SUD compared to AD without (63).

The relationship between BD and AUD is bidirectional. In younger patients, it appears that alcohol use and bipolar symptoms are more likely to increase or decrease in unison (64). Depression increases alcohol craving in BD patients with AUD. An exploratory sub-analysis (65) examined the impact of depressive symptoms on craving and drinking behavior in 30 comorbid patients participating in a 8-week, placebo-controlled relapse prevention study (acamprosate vs. placebo). The analyzed subgroup of bipolar patients was well-stabilized on different mood stabilizers (antipsychotics, antiepileptics, or lithium). Severity of depression correlated significantly with craving and drinking behavior 1 week later.

However, also the reverse is true (66), the pattern and frequency of AUD can foster new episodes of BD, both mania and depression (67, 68); increasing severity of AUD predicts occurrence of a new major depressive episode (MDE) (69). Co-occuring BD has a detrimental impact on subjects with AUD. The Collaborative Study on the Genetics of Alcoholism is a family pedigree investigation that enrolled treatment-seeking alcohol-dependent probands who met the DSM-IV criteria for alcohol dependence (70). Of the 228 Bipolar probands, 75.4% (74% in bipolar I patients and 77% in bipolar II patients) fulfilled criteria for DSM-IV life time alcohol dependence. Comparing 5-year prospective data of BP-I and -II probands with and without alcohol dependence confirmed (71), in line with previous retrospective studies (60), a more severe course of BD in comorbid bipolar I individuals, whereas bipolar II individuals were less severely impaired by comorbid alcohol use.

The sequence of onset of each respective disorder might be of importance for early detection and possibly treatment of persons on risk. In a study by Frank et al., substance use preceded in 60% but succeeded in 7% the first manic episode which favors SUD and AUD as a trigger for BD. In one third of cases the temporal sequence remained unclear (72). Analysis of data from the National Comorbidity Survey Replication study revealed that SUD starting in adolescence leads to an ~3-fold increased odds of subsequent mood disorders, especially BD (73). The fact that juvenile-onset BD is a risk factor for SUD was also replicated in other studies (74, 75). Other studies, however, are in support of BD as the primary disorder followed by SUD and/or AUD. Preisig et al. (76) concluded from their study on familial relationship between mood disorders and alcoholism that BD tends to precede AUD. Comparing retrospectively three samples of bipolar patients (group 1: BD without AUD and SUD, group 2: onset of BD precedes AUD and SUD, and group 3: onset of a SUD precedes BD), Feinman and colleagues found that subjects in the second group showed a significantly earlier onset of affective symptoms than those in the other two groups, also suggesting that BD is a breeding ground for AUD or SUD (77). Whether SUD or AUD onset prior to BD results in a more or less severe course of BD is still a matter of discussion. The study of Feinman and Dunner found higher rates of suicide attempts in their group 3 (SUD prior to BD), whereas Winokur et al. report on a milder course of BD in those with prior onset of SUD (78). The relationship between SUD or AUD and BD is probably not just bidirectional but more complex with several confounding variables. McElroy et al. (79), for example, retrospectively showed an association between early onset BD, mixed symptoms, psychiatric comorbidity and SUD. Thus, early detection of both BD and being on risk for SUD is essential to avoid disastrous outcomes (10), but further prospective research of the complex relationship in larger samples is still needed.

## Treatment Strategies in Comorbid BD and AUD—General Principles of Treatment

This chapter deals with the intermediate and long-term treatment of comorbid BD and AUD. We do not recap acute treatments for detoxification or delirium on one side, and mania or severe depression on the other side. These acute treatments are symptom-orientated, rarely different in comorbid vs. non-comorbid patients and depend on the predominant symptomatology (affective vs. addictive) that needs attention first. For intermediate and long-term treatment, the dogma persisted for a long time that AUD needs to be treated first and sufficiently before attention should be paid to the mental health disorder. Today, strategies that promote concomitant therapy of dual disorders are the established treatment of choice (80) and recommended in major guidelines (81). However, treatment adherence and compliance remain a challenge in this special group, since medications are often not taken as prescribed (61) and psychotherapy appointments are often missed. Studies support that the most important predictor of non-adherence in BD is comorbid alcohol and/or drug abuse (82, 83). Thus, effective psychosocial (84), psychoeducational (85, 86) or psychotherapeutic (87, 88) intervention for AUD and BD can also positively impact on medication adherence and, by this, ameliorate the course especially of BD (84).

Successful treatment of comorbid BD and AUD is a time-consuming process. Except from few specialized long-term inpatient settings for comorbid patients (89) the emphasis of all treatment concepts is on outpatient settings as behavioral changes and building up resilience is a long process in both disorders. As relapses and recurrences are rather the rule than the exception, regular outpatient contacts, emergency numbers to call in case of an imminent relapse and a timely and easy access to inpatient treatment for either one of the disorders are crucial. The German S3 Guidelines for AUD recommend that both disorders, BD and AUD, should be treated in one setting and by the same therapeutic team (49, 81). If not feasible, a close coordination of therapies, e.g., by means of a case manager, should be established.

## Psychotherapy

The evidence base for suitable psychotherapies in comorbid BD and AUD remains poor. The German S3 Guidelines for AUD (49) recommends cognitive behavioral therapy (CBT) as the best evidenced modality whereas there is no recommendation for other psychotherapies due to insufficient data.

This recommendation is, by large, based on the CBT studies conducted by Farren et al. In a prospective cohort study, 232 comorbid patients with alcohol dependence and an affective disorder (among whom 102 were individuals with BDs), received inpatient treatment with cognitive behavioral therapy for 4 weeks (90). The program also included psychoeducation on both disorders. At 6-month follow-up both groups (depressive and bipolar patients) showed a significant reduction of alcohol consumption, but no difference was found between patients with unipolar and bipolar disorder. At 5-year follow-up, there was still a significant long-term benefit, particularly in those who engaged in post-discharge supportive therapy. Early abstinence predicted later abstinence, and a significant number of those who reduced their drinking by 6 months also achieved complete abstinence after 5 years (91).

Other guidelines, e.g., the Canadian Network for Mood and Anxiety Treatments (CANMAT) do not recommend CBT but rather the integrated group therapy (IGT) developed by Weiss and colleagues which includes CBT and psychoeducation components. IGT has been studied in a pilot study (92) and 2 separate RCTs (93, 94) comparing it with either group drug counseling or no treatment. This manualized program with 20 weekly group sessions demonstrated effectiveness both for the prevention of alcohol and bipolar relapses (93) even at 8-month follow-up. IGT topics are identification of triggers preceding substance use, refusing drugs and alcohol, coping with BD without abusing substances, medication adherence, relationships with friends and family members, self-help group participation, weighing the pros and cons of recovery, and recognizing the warning signs of relapse, among other topics (95). A slimmed version with twelve sessions, developed by the same group, also demonstrated effectiveness (94).

The evidence for Assertive community treatment (AST) that has been examined in two RCTs is inconclusive, with one study showing a reduction of alcohol use, the other not when compared to standard clinical case management. Both studies included also patients with other major mental health disorders, such as MDD and schizophrenia; thus, both do not supply information exclusively about changes in the course of BD (96, 97). Only a follow-up evaluation of the first study after 3 years specifically reports about 51 patients with BD and comorbid SUD, stating that taking part in the AST program has also improved quality of life (QoL) and diverse functionality measures (98).

For contingency management and motivational therapy in comorbid BD and SUD, only low-level evidence exists, e.g., non-randomized, prospective studies, case series or retrospective studies. In the CANMAT guidelines they are only recommended as second-choice in situations where first choice treatments are not indicated or cannot be used, or when first-choice treatments have not worked (89).

In adolescents with comorbid BD and SUD, inclusion of the family appears crucial. Family-focused treatment (FFT) with psychoeducation is recommended and effective (99).

In summary, only few psychotherapeutic interventions have been studied in a randomized study design and mostly only by one research group. The most recent Cochrane review on psychotherapies of mental illness and comorbid SUDs examined 41 RCTs and concluded that it was impossible to rule in favor of any specific psychosocial treatment, because of a large array of methodological differences and difficulties impeding data pooling as well as interpretation (100).

## E-Mental Health Approaches

Not only in times of pandemics, but for the sake of high visibility, easy access and cost-effectiveness digital media are increasingly on the rise in health care, and have been used for screening and supplementing psychotherapy in affective disorders comorbid with AUD during recent years (101). For unipolar depression, efficacy for depressive symptoms as well as drinking behavior (cumulative duration of abstinence) was reported in a study comparing SMS twice daily (*n* = 26) vs. control condition (14-day “thank you” —SMS, *n* = 28). After 3 months of study completion, both Beck Depression Inventory scores and cumulative abstinence were significantly improved in the experimental group (102); however, the effect did not last (103). A subsequent, slightly larger study (*n* = 95) included comorbid patients both with unipolar and bipolar depression. Unfortunately, numbers and outcomes are not reported separately for unipolar and BD subjects. The study found a significant reduction in depression scores (*p* = 0.02) and perceived stress scores (*p* < 0.01) 3 months after completing a 30-day rehabilitation program in the intervention group. The intervention group- again receiving twice daily supportive text messages- also showed a significantly greater reduction in units per drinking day from baseline to 6 months after completion of the rehabilitation program. treatment point compared to the control group with a medium effect size (*P* = 0.03). At follow up 6 months after stopping text messaging (12 months after completion of the rehabilitation program) group differences in drinking or mood measures had vanished, again suggesting that the effect of text messaging was transient but not lasting.

The use or digital media and “blended care” is likely to increase in the future across treatment settings and will facilitate diagnosis and treatment of mental disorders including comorbid conditions. It's usefulness in BD patients comorbid with AUD, however, still needs to be further investigated.

## Pharmacological Options

Besides psychotherapy an individually tailored pharmacotherapy is essential in almost all BD patients with comorbid AUD. For BD, pharmacotherapy is an essential component to stabilize mood and prevent recurrences, whereas its role for treating AUD beyond controlling acute withdrawal symptoms is less clear. Randomized controlled studies in BD traditionally exclude patient with concurrent SUD. Thus, the evidence for choosing a mood stabilizer in BD with comorbid AUD is rather weak; strictly speaking, high levels evidence consists of altogether three placebo-controlled studies in this patient group (104–106). To make any suggestion (not even recommendations) about best available treatments we therefore rely on additional low-level evidence from open or retrospective studies and expert opinion.

In general, treatment-refractory patients are over-represented in the group of BD patients with comorbid SUD (107). As with most treatments, concurrent SUD including AUD is thus a predictor for inferior response to lithium. However, as shown in adolescents, achieving more mood stability with lithium can result in lower levels of alcohol or drug consumption (108). Positive effects of lithium on SUD apart from indirect effects via mood stabilization could not be substantiated so far (109).

Anticonvulsants, namely valproate, carbamazepine and lamotrigine, had been first line alternatives in BD to lithium for a long time. An open pilot study with valproate by Brady and colleagues in 1995 suggested a reduction of drinking days in parallel to a reduction of manic and depressive symptoms (110). Subsequently, Brady and colleagues conducted a 12-week, double-blind, placebo-controlled trial (105) in a small group of BD patients with comorbid AUD (*n* = 29). In this study, the valproate group had a significantly smaller percentage of subjects who relapsed to heavy drinking, but otherwise there were no significant differences in other alcohol-related outcomes. As far as BD symptomatology was concerned, there was only a significantly greater decrease in irritability. In the following, Salloum and colleagues conducted a 24-week, double-blind, placebo- controlled study with a slightly greater number of patients (*n* = 59). Valproate failed to demonstrate improvement in mood stabilization. The number of heavy drinking days, number of drinks per heavy drinking day, and serum biomarker levels of alcohol use in the valproate-treated group, however, were significantly reduced compared to the placebo group (106). Finally, the study by Kemp and colleagues in rapid-cycling (RC) BD patients with comorbid SUD (alcohol, cannabis or cocaine) consisted of an open label stabilization (up to 24 weeks) with all patients receiving lithium plus valproate, follow by a 6-month double- blind phase with randomization of stabilized patients to either lithium alone or lithium plus valproate. The study failed to demonstrate any effect of valproate on mood- related parameters (104). As far as AUD is concerned, the authors reported that of the 19 subjects with AUD, 58% no longer met criteria for active abuse or had entered into early full remission while in the open-label phase. Due to the very small number of subjects entering the double-blind phase (*n* = 31, corresponding to 21% of the original sample), there are no reliable data whether adding valproate to lithium is superior to lithium monotherapy, neither for mood-nor alcohol related outcomes.

For lamotrigine, only open-label evidence exists. In this small study (*n* = 28), lamotrigine improved mood symptoms, as well as decreased craving for alcohol and decreased carbohydrate deficient transferrin over 12 weeks (111). Unfortunately, so far, no confirmative controlled studies in BD + AUD with lamotrigine have been conducted (112). The only placebo-controlled study with lamotrigine in BD comorbid with SUD (cocaine) was negative across mood and substance related outcomes (113).

Carbamazepine has been traditionally used in acute alcohol withdrawal to reduce the risk of seizures and ameliorate physical symptoms. However, there are no reliable data whether it is of any usefulness in the long-term treatment of BD + AUD. Carbamazepine is metabolized by the liver and can, by itself, induce an increase in liver transaminases (ALAT, ASAT, γGT) and, in rare cases, cause liver failure. Thus, its use might put active alcohol users on risk.

A controlled study with topiramate in BD + AUD failed due to slow recruitment (114).

Atypical antipsychotics (aAP) have increasingly become a treatment of choice in BD. There was some expectation that their dopamine-stabilizing effects might also lead to a reduction of craving; however, a meta-analysis of the use of aAP as a group in primary AUD without comorbidities could not find any effects on drinking behavior or craving (115). The only exception was aripiprazole which reduced significantly number of drinks and heavy drinking days in one study (116).

Retrospective data suggested that, similar to aripiprazole (117), quetiapine might relieve alcohol graving in patients with BD and concomitant cocaine use (118). Subsequently, the same group conducted a double-blind, placebo-controlled study (119) in patients with BD + AUD. At the time of the study, 82% of subjects were in a depressive state. Quetiapine add-on to treatment as usual (TAU) had no effect on any alcohol-related outcomes, but produced a faster and significantly greater decrease of depressive symptoms. This finding is of note as many antidepressant treatment modalities are less effective in BD patients with comorbid AUD. The lack of efficacy of quetiapine against AUD was also confirmed in another placebo- controlled study (120). No controlled data for other aAP or antidepressants have, so far, been generated (see Table 1).

**Table 1:** Randomized controlled studies on pharmacological treatments of comorbid BD and AUD.

**References**	**Design**	**Intervention**	**Inclusion criteria**	**Alcohol or substance use disorders**	**Results**
Geller et al. (108)	RCT, DB	Lithium vs. PLC	12 BD-I; 5 BD- II disorder	Alcohol or substance dependence	Improvement of alcohol and SUD and affective symptoms
Brady et al. (105)	RCT, DB	Valproate vs. PLC			
Salloum et al. (106)	RCT, DB	Valproate vs. PLC (as add on to lithium)	59 BD-I disorder	Alcohol dependence	Improvement of AUD but not affective symptoms
Kemp et al. (104)	RCT, DB	Lithium + PLC vs. lithium + valproate after up to 6 months open label stabilization with lithium + valproate	149 BD I or II subjects with RC. *Post-hoc* analysis of those with AUD from a larger RC-BD sample.	Alcohol, cannabis, or cocaine abuse within the last 3 months or dependence within the last 6 months	Only 31 of 149 subjects could be stabilized on open label lithium + valproate. No difference between treatments in mood outcomes at the end of the 6-month DB phase. For alcohol related outcomes, more subjects improved with the lithium + valproate combination
Brown et al. (119)	RCT, DB	Quetiapine vs. PLC	102 BD-I disorder	Alcohol dependence	Improvement of affective but not alcohol use symptoms
Brown et al. (121)	RCT, DB	Naltrexon vs. PLC add on to cognitive therapy	50 BD-I; 10 BD-II; 2 BD-disorder not otherwise specified	Alcohol dependence	Improvement of AUD but not affective symptoms
Stedman et al. (120)	RCT, DB	Quetiapine vs. PLC as add on to lithium or valproate	362 BD-I disorder	Alcohol dependence	No influence of both alcohol use and affective symptoms
Tolliver et al. (122)	RCT, DB	Various affective relapse prevention medication; Acamprosat vs. PLC as add on	33 BD-I and II disorder	Alcohol dependence	No improvement of alcohol use frequency and amount, no influence on affective symptoms
Brown et al. (123)	RCT, DB	12 weeks of sustained release quetiapine (to 600 mg/d) add-on therapy vs. PLC	90 outpatients with BD-I or II disorders, depressed or mixed mood state	Alcohol dependence	No significant between-group differences were observed on the primary outcome measure of drinks per day or other alcohol-related or mood measures (*p* > 0.05). Overall side effect burden, glucose, and cholesterol were similar in the 2 groups
Brown et al. (124)	RCT, DB	12 weeks of ondansetron vs. PLC add-on to TAU	70 outpatients with BD-spectrum disorders	Early onset AUD (≤25 y)	No significant between-group differences in alcohol use measures were observed. A significant reduction in HRSD scores was observed (*p* = 0.045)

*The table illustrates the results of a systematic PubMed search on January 15, 2021, covering the years 1970–2020, using the following MeSH terms: alcohol abuse or alcohol dependence or alcohol use disorder and bipolar disorder or mania or manic depression and Randomized Controlled Trial. We also checked the literature lists of major textbooks on bipolar disorder for additional references*.

*AUD, Alcohol use disorder; BD, Bipolar disorder; DB, double-blind; HRSD, Hamilton Rating scale for Depression; PLC, Placebo; RC, Rapid cycling; RCT, randomized controlled trial; TAU, Treatment as usual*.

Limited data exist on the effect of anti-craving medication in AUD with comorbid BD. Results of an open study suggested a reduction of both craving and stabilization of mood with naltrexone in patients with BD + AUD (125). However, improvement of mood was not confirmed in a double-blind study with naltrexone add-on to cognitive behavioral therapy, and there was only a trend toward less alcohol consumption (121). Similar disappointing results have been reported from a controlled study with acamprosate in BD + AUD (122).

A recent experimental approach used ondansetron. Ondansetron is a 5-HT_3_ receptor antagonist used to prevent nausea and vomiting caused by chemo- or radiation therapy. A controlled study suggested a reduction of alcohol consumption with ondansetron (126). However, in a randomized, double-blind, placebo-controlled trial in outpatients with BD and early onset AUD (124) no reduction of alcohol use measures were observed; but interestingly, ondansetron led to an improvement of depressive symptomatology measured with the Hamilton Depression rating scale [HDRS (127)].

Table 1 supplies an overview of double-blind, randomized pharmacological studies for comorbid bipolar affective and AUDs, based on a systematic PubMed search.

## Conclusion

**Figure 1:**
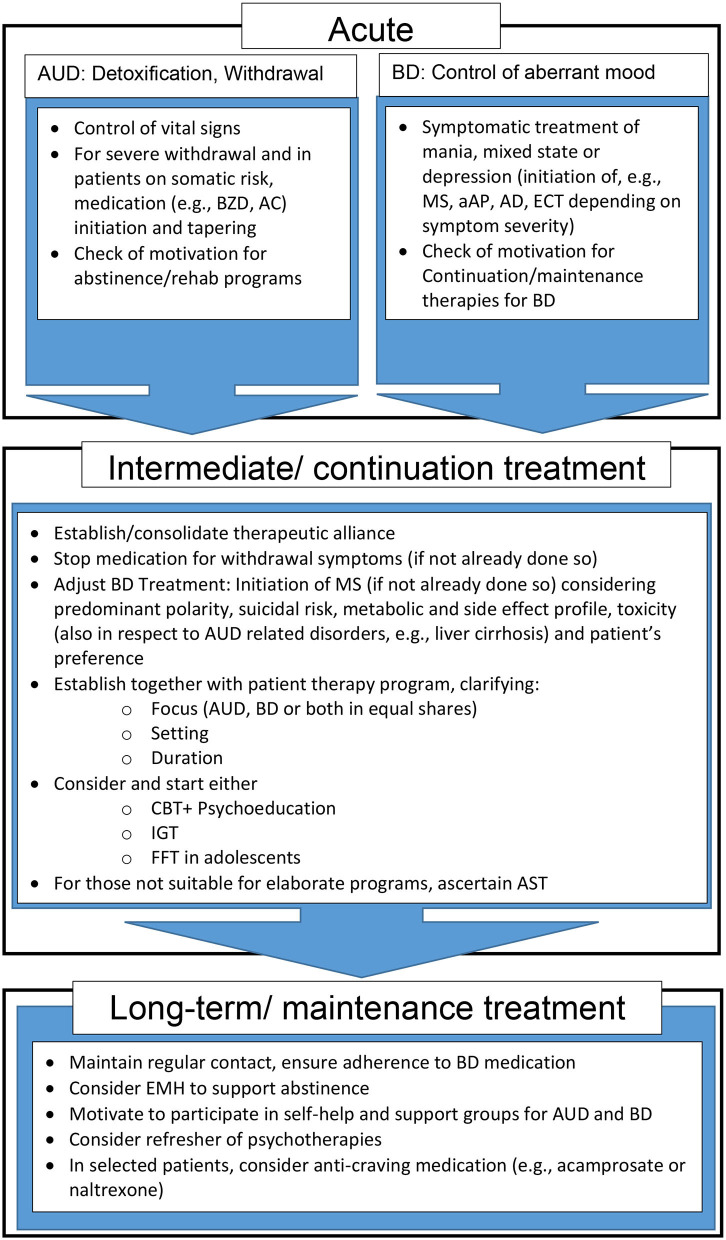
Proposed treatment and support algorithm for patients with comorbid AUD and BD. aAP, atypical antipsychotics; AC, Anticonvulsants; AD, Antidepressants; AST, Assertive community treatment; AUD, Alcohol use disorder; BD, Bipolar Disorder; BZD, benzodiazepines; CBT, Cognitive behavioral therapy; ECT, Electroconvulsive therapy; EMH, E-Mental Health; FFT, Family-focused therapy; IGT, Integrated group therapy; MS, Mood stabilizer.

In BD, comorbid SUD and especially AUD are rather the rule than the exception. Pharmacological and integrated psychotherapeutic approaches that give equal weight to both disorders, while still scarce, are recommended. CBT and IGT have the best, but still insufficient evidence- base as psychosocial treatments. Figure 1 depicts a proposed therapy algorithm based on the evidence presented in this article. Supportive pharmacotherapy should be mainly centered around BD, with mood stabilizer, e.g., lithium and valproate, still the treatment of choice. However, there is clearly more research needed to develop reliable treatment algorithms for comorbid BD and AUD.

## Author Contributions

All authors designed the work, conducted the necessary literature search, drafted the manuscript, provide approval for publication, and agree to be accountable for all aspects of the work.

## Conflict of Interest

The authors declare that the research was conducted in the absence of any commercial or financial relationships that could be construed as a potential conflict of interest.

## References

[1] Angst J. Bipolar disorder–methodological problems and future perspectives. Dialogues Clin Neurosci. (2008) 10:129–39. 10.31887/DCNS.2008.10.2/jangst 18689284 PMC3181871

[2] Merikangas KR Jin R He JP Kessler RC Lee S Sampson NA. Prevalence and correlates of bipolar spectrum disorder in the world mental health survey initiative. Arch Gen Psychiatry. (2011) 68:241–51. 10.1001/archgenpsychiatry.2011.12 21383262 PMC3486639

[3] Kessler RC Andrews G Mroczek D Ustun B Wittchen H-U. The world health organization composite international diagnostic interview short-form (CIDI-SF). Int J Methods Psychiatr Res. (1998) 7:171–85. 10.1002/mpr.47

[4] Jacobi F Höfler M Strehle J Mack S Gerschler A Scholl L. Psychische Störungen in der Allgemeinbevölkerung: Studie zur Gesundheit Erwachsener in Deutschland und ihr Zusatzmodul Psychische Gesundheit (DEGS1-MH). Nervenarzt. (2014) 85:77–87. 10.1007/s00115-013-3961-y 24441882

[5] Jacobi F Klose M Wittchen HU. Psychische Störungen in der deutschen Allgemeinbevölkerung: Inanspruchnahme von Gesundheitsleistungen und Ausfalltage. BundesgesundheitsblattGesundheitsforschungGesundheitsschutz. (2004) 47:736–44. 10.1007/s00103-004-0885-5 15340716

[6] Merikangas KR Akiskal HS Angst J Greenberg PE Hirschfeld RM Petukhova M. Lifetime and 12-month prevalence of bipolar spectrum disorder in the national comorbidity survey replication. ArchGenPsychiatry. (2007) 64:543–52. 10.1001/archpsyc.64.5.543 17485606 PMC1931566

[7] Pinto JV Medeiros LS Santana da Rosa G Santana de Oliveira CE Crippa JAS Passos IC. The prevalence and clinical correlates of cannabis use and cannabis use disorder among patients with bipolar disorder: a systematic review with meta-analysis and meta-regression. Neurosci Biobehav Rev. (2019) 101:78–84. 10.1016/j.neubiorev.2019.04.004 30974123

[8] Amin-Esmaeili M Rahimi-Movaghar A Sharifi V Hajebi A Mojtabai R Radgoodarzi R. Alcohol use disorders in Iran: prevalence, symptoms, correlates, and comorbidity. Drug Alcohol Depend. (2017) 176:48–54. 10.1016/j.drugalcdep.2017.02.018 28514696

[9] Grant BF Goldstein RB Saha TD Chou SP Jung J Zhang H. Epidemiology of DSM-5 alcohol use disorder: results from the national epidemiologic survey on alcohol and related conditions III. JAMA Psychiatry. (2015) 72:757–66. 10.1001/jamapsychiatry.2015.0584 26039070 PMC5240584

[10] Joshi G Wilens T. Comorbidity in pediatric bipolar disorder. Child Adolesc Psychiatr Clin N Am. (2009) 18:291–319, vii–viii. 10.1016/j.chc.2008.12.005 19264265 PMC3757953

[11] American Psychiatric Association. Diagnostic and Statistical Manual of Mental Disorders. Washington, DC: APA Press (2013).

[12] Kranzler HR Soyka M. Diagnosis and pharmacotherapy of alcohol use disorder: a review. JAMA. (2018) 320:815–24. 10.1001/jama.2018.11406 30167705 PMC7391072

[13] Grunze A Born C Fredskild MU Grunze H. How does adding the DSM-5 criterion increased energy/activity for mania change the bipolar landscape? Front Psychiatry. (2021) 12:e638440. 10.3389/fpsyt.2021.638440 33679488 PMC7930230

[14] Fredskild MU Mintz J Frye MA McElroy SL Nolen WA Kupka R. Adding increased energy or activity to criterion (A) of the DSM-5 definition of hypomania and mania: effect on the diagnoses of 907 patients from the bipolar collaborative network. J Clin Psychiatry. (2019) 80:19m12834. 10.4088/JCP.19m12834 31665571

[15] Ferrari AJ Stockings E Khoo JP Erskine HE Degenhardt L Vos T. The prevalence and burden of bipolar disorder: findings from the global burden of disease study 2013. Bipolar Disord. (2016) 18:440–50. 10.1111/bdi.12423 27566286

[16] Ugochukwu C Bagot KS Delaloye S Pi S Vien L Garvey T. The importance of quality of life in patients with alcohol abuse and dependence. Harv Rev Psychiatry. (2013) 21:1–17. 10.1097/HRP.0b013e31827fd8aa 23656759

[17] Levola J Kaskela T Holopainen A Sabariego C Tourunen J Cieza A. Psychosocial difficulties in alcohol dependence: a systematic review of activity limitations and participation restrictions. Disabil Rehabil. (2014) 36:1227–39. 10.3109/09638288.2013.837104 24079366

[18] Young AH Grunze H. Physical health of patients with bipolar disorder. Acta Psychiatr Scand Suppl. (2013) 442:3–10. 10.1111/acps.12117 23581787

[19] Whiteford HA Degenhardt L Rehm J Baxter AJ Ferrari AJ Erskine HE. Global burden of disease attributable to mental and substance use disorders: findings from the global burden of disease study 2010. Lancet. (2013) 382:1575–86. 10.1016/S0140-6736(13)61611-6 23993280

[20] GBD Study Group. Global, regional, and national incidence, prevalence, and years lived with disability for 354 diseases and injuries for 195 countries and territories, 1990-2017: a systematic analysis for the global burden of disease study 2017. Lancet. (2018) 392:1789–858. 10.1016/s0140-6736(18)32279-7 30496104 PMC6227754

[21] Hunt GE Malhi GS Cleary M Lai HM Sitharthan T. Prevalence of comorbid bipolar and substance use disorders in clinical settings, 1990-2015: Systematic review and meta-analysis. J Affect Disord. (2016) 206:331–49. 10.1016/j.jad.2016.07.011 27476137

[22] Strakowski SM McElroy SL Keck PE West SA. The effects of antecedent substance abuse on the development of first-episode psychotic mania. J Psychiatr Res. (1996) 30:59–68. 10.1016/0022-3956(95)00044-5 8736468

[23] Cardoso BM Kauer SAM Dias VV Andreazza AC Cereser KM Kapczinski F. The impact of co-morbid alcohol use disorder in bipolar patients. Alcohol. (2008) 42:451–7. 10.1016/j.alcohol.2008.05.003 18760714

[24] Oquendo MA Currier D Liu SM Hasin DS Grant BF Blanco C. Increased risk for suicidal behavior in comorbid bipolar disorder and alcohol use disorders: results from the National Epidemiologic Survey on Alcohol and Related Conditions (NESARC). J Clin Psychiatry. (2010) 71:902–9. 10.4088/JCP.09m05198gry 20667292 PMC2914308

[25] Yoon YH Chen CM Yi HY Moss HB. Effect of comorbid alcohol and drug use disorders on premature death among unipolar and bipolar disorder decedents in the United States, 1999 to 2006. Compr Psychiatry. (2011) 52:453–64. 10.1016/j.comppsych.2010.10.005 21146814 PMC3139776

[26] Regier DA Farmer ME Rae DS. Comorbidity of mental disorders with alcohol and other drug abuse: results from the epidemiologic catchment area (ECA) study. JAMA. (1990) 264:2511–8. 2232018

[27] Cerullo MA Strakowski SM. The prevalence and significance of substance use disorders in bipolar type I and II disorder. Subst Abuse Treat Prev Policy. (2007) 2:29. 10.1186/1747-597X-2-29 17908301 PMC2094705

[28] Hunt GE Malhi GS Cleary M Lai HM Sitharthan T. Comorbidity of bipolar and substance use disorders in national surveys of general populations, 1990-2015: systematic review and meta-analysis. J Affect Disord. (2016) 206:321–30. 10.1016/j.jad.2016.06.051 27426694

[29] Lucatch AM Coles AS Hill KP George TP. Cannabis and mood disorders. Curr Addict Rep. (2018) 5:336–45. 10.1007/s40429-018-0214-y 30643708 PMC6329464

[30] Ostacher MJ Perlis RH Nierenberg AA Calabrese J Stange JP Salloum I. Impact of substance use disorders on recovery from episodes of depression in bipolar disorder patients: prospective data from the systematic treatment enhancement program for bipolar disorder (STEP-BD). Am J Psychiatry. (2010) 167:289–97. 10.1176/appi.ajp.2009.09020299 20008948 PMC2918249

[31] McElroy SL Altshuler LL Suppes T Keck PE Frye MA Denicoff KD. Axis I psychiatric comorbidity and its relationship to historical illness variables in 288 patients with bipolar disorder. Am J Psychiatry. (2001) 158:420–6. 10.1176/appi.ajp.158.3.420 11229983

[32] Frye MA Altshuler LL McElroy SL Suppes T Keck PE Denicoff K. Gender differences in prevalence, risk, and clinical correlates of alcoholism comorbidity in bipolar disorder. Am J Psychiatry. (2003) 160:883–9. 10.1176/appi.ajp.160.5.883 12727691

[33] Levander E Frye MA McElroy S Suppes T Grunze H Nolen WA. Alcoholism and anxiety in bipolar illness: differential lifetime anxiety comorbidity in bipolar I women with and without alcoholism. J Affect Disord. (2007) 101:211–7. 10.1016/j.jad.2006 17254638

[34] Kessler RC Nelson CB McGonagle KA Liu J Swartz M Blazer DG. Comorbidity of DSM-III-R major depressive disorder in the general population: results from the US National Comorbidity Survey. Br J Psychiatry Suppl. (1996) 30:17–30. 8864145

[35] Zamora-Rodriguez FJ Sanchez-Waisen-Hernandez MR Guisado-Macias JA Vaz-Leal FJ. Substance use and course of bipolar disorder in an inpatient sample. Actas Esp Psiquiatr. (2018) 46:183–91. 30338775

[36] Scott J Grunze H Meyer TD Nendick J Watkins H Ferrier N. A bipolar II cohort (ABC): the association of functional disability with gender and rapid cycling. J Affect Disord. (2015) 185:204–8. 10.1016/j.jad.2015.06.050 26209962

[37] Dittmann S Biedermann NC Grunze H Hummel B Schärer LO Kleindienst N. The Stanley foundation bipolar network: results of the naturalistic follow-up study after 2.5 years of follow-up in the German centres. Neuropsychobiology. (2002) 46(Suppl. 1):2–9. 10.1159/000068018 12571425

[38] Post RM Altshuler L Kupka R McElroy S Frye MA Rowe M. More pernicious course of bipolar disorder in the United States than in many European countries: implications for policy and treatment. J Affect Disord. (2014) 160:27–33. 10.1016/j.jad.2014.02.006 24709019

[39] Kessler RC McGonagle KA Zhao S Nelson CB Hughes M Eshleman S. Lifetime and 12-month prevalence of DSM-III-R psychiatric disorders in the United States. Results from the National Comorbidity Survey. Arch Gen Psychiatry. (1994) 51:8–19. 10.1001/archpsyc.1994.03950010008002 8279933

[40] Nolen WA Luckenbaugh DA Altshuler LL Suppes T McElroy SL Frye MA. Correlates of 1-year prospective outcome in bipolar disorder: results from the Stanley Foundation Bipolar Network. Am J Psychiatry. (2004) 161:1447–54. 10.1176/appi.ajp.161.8.1447 15285972

[41] Post RM Altshuler L Kupka R McElroy SL Frye MA Rowe M. Multigenerational positive family history of psychiatric disorders is associated with a poor prognosis in bipolar disorder. J Neuropsychiatry Clin Neurosci. (2015) 27:304–10. 10.1176/appi.neuropsych.14080204 26258489

[42] Haro G Calabrese JR Larsson C Shirley ER Martin E Leal C. The relationship of personality traits to substance abuse in patients with bipolar disorder. Eur Psychiatry. (2007) 22:305–8. 10.1016/j.eurpsy.2007.03.009 17521889

[43] Berrettini WH. Genetics of psychiatric disease. Annu Rev Med. (2000) 51:465–79. 10.1146/annurev.med.51.1.465 10774477

[44] Chang YH Lee SY Wang TY Chen SL Tzeng NS Chen PS. Comorbid alcohol dependence disorder may be related to aldehyde dehydrogenase 2 (ALDH2) and alcohol dehydrogenase 1B (ADH1B) in bipolar II disorder, but only to ALDH2 in bipolar I disorder, in Han Chinese. Bipolar Disord. (2015) 17:536–42. 10.1111/bdi.12313 26033520

[45] Yasseen B Kennedy JL Zawertailo LA Busto UE. Comorbidity between bipolar disorder and alcohol use disorder: association of dopamine and serotonin gene polymorphisms. Psychiatry Res. (2010) 176:30–3. 10.1016/j.psychres.2008.12.009 20071033

[46] Schellekens AF Franke B Ellenbroek B Cools A de Jong CA Buitelaar JK. Reduced dopamine receptor sensitivity as an intermediate phenotype in alcohol dependence and the role of the COMT Val158Met and DRD2 Taq1A genotypes. Arch Gen Psychiatry. (2012) 69:339–48. 10.1001/archgenpsychiatry.2011.1335 22474103

[47] Jang SK Saunders G Liu M Jiang Y Liu DJ Vrieze S. Genetic correlation, pleiotropy, and causal associations between substance use and psychiatric disorder. Psychol Med. (2020). 10.1017/s003329172000272x. [Epub ahead of print]. 32762793 PMC8759148

[48] Brown SE Suppes T Adinoff B Rajan TN. Drug abuse and bipolar disorder: comorbidity or misdiagnosis? J Affect Disord. (2001) 65:105–15. 10.1016/s0165-0327(00)00169-5 11356233

[49] Deutsche Gesellschaft für Suchtforschung und Suchttherapie e V. AWMF S3 Leitline Screening, Diagnose und Behandlung alkoholbezogener Störungen. Aktualisierte Version 2020 (2020). Available online at: https://www.awmf.org/leitlinien/detail/ll/076-001.html (accessed February 2, 2021).

[50] Salloum IM Brown ES. Management of comorbid bipolar disorder and substance use disorders. Am J Drug Alcohol Abuse. (2017) 43:366–76. 10.1080/00952990.2017.1292279 28301219

[51] Allen JP Litten RZ Fertig JB Babor T. A review of research on the alcohol use disorders identification test (AUDIT). Alcohol Clin Exp Res. (1997) 21:613–9. 9194913

[52] Reinert DF Allen JP. The alcohol use disorders identification test (AUDIT): a review of recent research. Alcohol Clin Exp Res. (2002) 26:272–9. 11964568

[53] Preuss UW Gouzoulis-Mayfrank E Havemann-Reinecke U Schäfer I Beutel M Hoch E. Psychiatric comorbidity in alcohol use disorders: results from the German S3 guidelines. Eur Arch Psychiatry Clin Neurosci. (2018) 268:219–29. 10.1007/s00406-017-0801-2 28439723

[54] McKowen JW Frye MA Altshuler LL Gitlin MJ. Patterns of alcohol consumption in bipolar patients comorbid for alcohol abuse or dependence. Bipolar Disord. (2005) 7:377–81. 10.1111/j.1399-5618.2005.00208.x 16026491

[55] Hoff RA Rosenheck RA. The cost of treating substance abuse patients with and without comorbid psychiatric disorders. Psychiatr Serv. (1999) 50:1309–15. 10.1176/ps.50.10.1309 10506299

[56] Alonso J Petukhova M Vilagut G Chatterji S Heeringa S Ustun TB. Days out of role due to common physical and mental conditions: results from the WHO World Mental Health surveys. Mol Psychiatry. (2011) 16:1234–46. 10.1038/mp.2010.101 20938433 PMC3223313

[57] Gore FM Bloem PJ Patton GC Ferguson J Joseph V Coffey C. Global burden of disease in young people aged 10-24 years: a systematic analysis. Lancet. (2011) 377:2093–102. 10.1016/S0140-6736(11)60512-6 21652063

[58] Altshuler LL Kupka RW Hellemann G Frye MA Sugar CA McElroy SL. Gender and depressive symptoms in 711 patients with bipolar disorder evaluated prospectively in the Stanley Foundation Bipolar Treatment Outcome Network. Am J Psychiatry. (2010) 167:708–15. 10.1176/appi.ajp.2009.09010105 20231325

[59] Greenfield SF Pettinati HM O'Malley S Randall PK Randall CL. Gender differences in alcohol treatment: an analysis of outcome from the COMBINE study. Alcohol Clin Exp Res. (2010) 34:1803–12. 10.1111/j.1530-0277.2010.01267.x 20645934 PMC3048309

[60] Rakofsky JJ Dunlop BW. Do alcohol use disorders destabilize the course of bipolar disorder? J Affect Disord. (2013) 145:1–10. 10.1016/j.jad.2012.06.012 22858208

[61] Manwani SG Szilagyi KA Zablotsky B Hennen J Griffin ML Weiss RD. Adherence to pharmacotherapy in bipolar disorder patients with and without co-occurring substance use disorders. J Clin Psychiatry. (2007) 68:1172–6. 10.4088/jcp.v68n0802 17854240

[62] Soyka M. Substance misuse, psychiatric disorder and violent and disturbed behaviour. Br J Psychiatry. (2000) 176:345–50. 10.1192/bjp.176.4.345 10827882

[63] Modestin J Wuermle O. Criminality in men with major mental disorder with and without comorbid substance abuse. Psychiatry Clin Neurosci. (2005) 59:25–9. 10.1111/j.1440-1819.2005.01327.x 15679536

[64] Fleck DE Arndt S DelBello MP Strakowski SM. Concurrent tracking of alcohol use and bipolar disorder symptoms. Bipolar Disord. (2006) 8:338–44. 10.1111/j.1399-5618.2006.00332.x 16879134

[65] Prisciandaro JJ Desantis SM Chiuzan C Brown DG Brady KT Tolliver BK. Impact of depressive symptoms on future alcohol use in patients with co-occurring bipolar disorder and alcohol dependence: a prospective analysis in an 8-week randomized controlled trial of acamprosate. Alcohol Clin Exp Res. (2012) 36:490–6. 10.1111/j.1530-0277.2011.01645.x 21933201 PMC3248624

[66] Salloum IM Thase ME. Impact of substance abuse on the course and treatment of bipolar disorder. Bipolar Disord. (2000) 2:269–80. 10.1034/j.1399-5618.2000.20308.x 11249805

[67] Grant BF Stinson FS Dawson DA Chou SP Dufour MC Compton W. Prevalence and co-occurrence of substance use disorders and independent mood and anxiety disorders: results from the National Epidemiologic Survey on Alcohol and Related Conditions. Arch Gen Psychiatry. (2004) 61:807–16. 10.1001/archpsyc.61.8.807 15289279

[68] Conway KP Compton W Stinson FS Grant BF. Lifetime comorbidity of DSM-IV mood and anxiety disorders and specific drug use disorders: results from the National Epidemiologic Survey on Alcohol and Related Conditions. J Clin Psychiatry. (2006) 67:247–57. 10.4088/jcp.v67n0211 16566620

[69] Jaffee WB Griffin ML Gallop R Meade CS Graff F Bender RE. Depression precipitated by alcohol use in patients with co-occurring bipolar and substance use disorders. J Clin Psychiatry. (2009) 70:171–6. 10.4088/jcp.08m04011 19192456 PMC2713748

[70] Begleiter H Reich T Nurnberger J Jr Li TK Conneally PM Edenberg H. Description of the genetic analysis workshop 11 collaborative study on the genetics of alcoholism. Genet Epidemiol. (1999) 17(Suppl. 1):S25–30. 10.1002/gepi.1370170705 10597407

[71] Preuss UW Hesselbrock MN Hesselbrock VM. A prospective comparison of bipolar I and II subjects with and without comorbid alcohol dependence from the COGA dataset. Front Psychiatry. (2020) 11:522228. 10.3389/fpsyt.2020.522228 33408647 PMC7779525

[72] Frank E Boland E Novick DM Bizzarri JV Rucci P. Association between illicit drug and alcohol use and first manic episode. Pharmacol Biochem Behav. (2007) 86:395–400. 10.1016/j.pbb.2006 17188743 PMC1876823

[73] Kenneson A Funderburk JS Maisto SA. Substance use disorders increase the odds of subsequent mood disorders. Drug Alcohol Depend. (2013) 133:338–43. 10.1016/j.drugalcdep.2013.06.011 23906994

[74] Wilens TE Biederman J Millstein RB Wozniak J Hahesy AL Spencer TJ. Risk for substance use disorders in youths with child- and adolescent-onset bipolar disorder. J Am Acad Child Adolesc Psychiatry. (1999) 38:680–5. 10.1097/00004583-199906000-00014 10361785

[75] Kenneson A Funderburk JS Maisto SA. Risk factors for secondary substance use disorders in people with childhood and adolescent-onset bipolar disorder: opportunities for prevention. Compr Psychiatry. (2013) 54:439–46. 10.1016/j.comppsych.2012.12.008 23332720

[76] Preisig M Fenton BT Stevens DE Merikangas KR. Familial relationship between mood disorders and alcoholism. Compr Psychiatry. (2001) 42:87–95. 10.1053/comp.2001.21221 11244143

[77] Feinman JA Dunner DL. The effect of alcohol and substance abuse on the course of bipolar affective disorder. J Affect Disord. (1996) 37:43–9. 10.1016/0165-0327(95)00080-1 8682977

[78] Winokur G Coryell W Akiskal HS Maser JD Keller MB Endicott J. Alcoholism in manic-depressive (bipolar) illness: familial illness, course of illness, and the primary-secondary distinction. Am J Psychiatry. (1995) 152:365–72. 10.1176/ajp.152.3.365 7864261

[79] McElroy SL Strakowski SM Keck PE Jr Tugrul KL West SA Lonczak HS. Differences and similarities in mixed and pure mania. Compr Psychiatry. (1995) 36:187–94. 10.1016/0010-440x(95)90080-f 7648841

[80] Brady KT Verduin ML Tolliver BK. Treatment of patients comorbid for addiction and other psychiatric disorders. Curr Psychiatry Rep. (2007) 9:374–80. 10.1007/s11920-007-0048-0 17915076

[81] Mann K Batra A Fauth-Bühler M Hoch E. German guidelines on screening, diagnosis and treatment of alcohol use disorders. Eur Addict Res. (2017) 23:45–60. 10.1159/000455841 28178695

[82] Lingam R Scott J. Treatment non-adherence in affective disorders. Acta Psychiatr Scand. (2002) 105:164–72. 10.1034/j.1600-0447.2002.1r084.x 11939969

[83] Arvilommi P Suominen K Mantere O Leppämäki S Valtonen H Isometsä E. Predictors of adherence to psychopharmacological and psychosocial treatment in bipolar I or II disorders—an 18-month prospective study. J Affect Disord. (2014) 155:110–7. 10.1016/j.jad.2013.10.032 24262639

[84] Gaudiano BA Weinstock LM Miller IW. Improving treatment adherence in patients with bipolar disorder and substance abuse: rationale and initial development of a novel psychosocial approach. J Psychiatr Pract. (2011) 17:5–20. 10.1097/01.pra.0000393840.18099.d6 21266890 PMC3071706

[85] Clarkin JF Carpenter D Hull J Wilner P Glick I. Effects of psychoeducational intervention for married patients with bipolar disorder and their spouses. Psychiatr Serv. (1998) 49:531–3. 10.1176/ps.49.4.531 9550248

[86] Colom F Vieta E Martinez-Aran A Reinares M Goikolea JM Benabarre A. A randomized trial on the efficacy of group psychoeducation in the prophylaxis of recurrences in bipolar patients whose disease is in remission. Arch Gen Psychiatry. (2003) 60:402–7. 10.1001/archpsyc.60.4.402 12695318

[87] Lam DH Watkins ER Hayward P Bright J Wright K Kerr N. A randomized controlled study of cognitive therapy for relapse prevention for bipolar affective disorder: outcome of the first year. Arch Gen Psychiatry. (2003) 60:145–52. 10.1001/archpsyc.60.2.145 12578431

[88] Lam D Bright J Jones S Hayward P Schuck N Chisholm D. Cognitive therapy for bipolar illness—A pilot study of relapse prevention. Cogn Ther Res. (2000) 24:503–20. 10.1023/A:1005557911051

[89] Beaulieu S Saury S Sareen J Tremblay J Schütz CG McIntyre RS. The Canadian network for mood and anxiety treatments (CANMAT) task force recommendations for the management of patients with mood disorders and comorbid substance use disorders. Ann Clin Psychiatry. (2012) 24:38–55. 22303521

[90] Farren CK Mc Elroy S. Treatment response of bipolar and unipolar alcoholics to an inpatient dual diagnosis program. J Affect Disord. (2008) 106:265–72. 10.1016/j.jad.2007.07.006 17707085

[91] Farren CK Murphy P McElroy S. A 5-year follow-up of depressed and bipolar patients with alcohol use disorder in an Irish population. Alcohol Clin Exp Res. (2014) 38:1049–58. 10.1111/acer.12330 24428168

[92] Weiss RD Griffin ML Greenfield SF Najavits LM Wyner D Soto JA. Group therapy for patients with bipolar disorder and substance dependence: results of a pilot study. J Clin Psychiatry. (2000) 61:361–7. 10.4088/jcp.v61n0507 10847311

[93] Weiss RD Griffin ML Kolodziej ME Greenfield SF Najavits LM Daley DC. A randomized trial of integrated group therapy versus group drug counseling for patients with bipolar disorder and substance dependence. Am J Psychiatry. (2007) 164:100–7. 10.1176/ajp.2007.164.1.100 17202550

[94] Weiss RD Griffin ML Jaffee WB Bender RE Graff FS Gallop RJ. A “community-friendly” version of integrated group therapy for patients with bipolar disorder and substance dependence: a randomized controlled trial. Drug Alcohol Depend. (2009) 104:212–9. 10.1016/j.drugalcdep.2009.04.018 19573999 PMC2735139

[95] Gold AK Otto MW Deckersbach T Sylvia LG Nierenberg AA Kinrys G. Substance use comorbidity in bipolar disorder: a qualitative review of treatment strategies and outcomes. Am J Addict. (2018) 27:188–201. 10.1111/ajad.12713 29596721

[96] Drake RE McHugo GJ Clark RE Teague GB Xie H Miles K. Assertive community treatment for patients with co-occurring severe mental illness and substance use disorder: a clinical trial. Am J Orthopsychiatry. (1998) 68:201–15. 10.1037/h0080330 9589759

[97] Essock SM Mueser KT Drake RE Covell NH McHugo GJ Frisman LK. Comparison of ACT and standard case management for delivering integrated treatment for co-occurring disorders. Psychiatr Serv. (2006) 57:185–96. 10.1176/appi.ps.57.2.185 16452695

[98] Drake RE Xie H McHugo GJ Shumway M. Three-year outcomes of long-term patients with co-occurring bipolar and substance use disorders. Biol Psychiatry. (2004) 56:749–56. 10.1016/j.biopsych.2004.08.020 15556119

[99] Miklowitz DJ. Family treatment for bipolar disorder and substance abuse in late adolescence. J Clin Psychol. (2012) 68:502–13. 10.1002/jclp.21855 22504610 PMC3872485

[100] Hunt GE Siegfried N Morley K Brooke-Sumner C Cleary M. Psychosocial interventions for people with both severe mental illness and substance misuse. Cochrane Database Syst Rev. (2019) 12:Cd001088. 10.1002/14651858.CD001088.pub4 31829430 PMC6906736

[101] Gustafson DH Boyle MG Shaw BR Isham A McTavish F Richards S. An e-health solution for people with alcohol problems. Alcohol Res Health. (2011) 33:327–37. 23293549 PMC3536059

[102] Agyapong VI Ahern S McLoughlin DM Farren CK. Supportive text messaging for depression and comorbid alcohol use disorder: single-blind randomised trial. J Affect Disord. (2012) 141:168–76. 10.1016/j.jad.2012.02.040 22464008

[103] Agyapong VI McLoughlin DM Farren CK. Six-months outcomes of a randomised trial of supportive text messaging for depression and comorbid alcohol use disorder. J Affect Disord. (2013) 151:100–4. 10.1016/j.jad.2013.05.058 23800443

[104] Kemp DE Gao K Ganocy SJ Elhaj O Bilali SR Conroy C. A 6-month, double-blind, maintenance trial of lithium monotherapy versus the combination of lithium and divalproex for rapid-cycling bipolar disorder and co-occurring substance abuse or dependence. J Clin Psychiatry. (2009) 70:113–21. 10.4088/jcp.07m04022 19192457 PMC3587136

[105] Brady KT Myrick H Henderson S Coffey SF. The use of divalproex in alcohol relapse prevention: a pilot study. Drug Alcohol Depend. (2002) 67:323–30. 10.1016/s0376-8716(02)00105-9 12127203

[106] Salloum IM Cornelius JR Daley DC Kirisci L Himmelhoch JM Thase ME. Efficacy of valproate maintenance in patients with bipolar disorder and alcoholism: a double-blind placebo-controlled study. Arch Gen Psychiatry. (2005) 62:37–45. 10.1001/archpsyc.62.1.37 15630071

[107] Goldberg JF Garno JL Leon AC Kocsis JH Portera L. A history of substance abuse complicates remission from acute mania in bipolar disorder. J Clin Psychiatry. (1999) 60:733–40. 10.4088/jcp.v60n1103 10584760

[108] Geller B Cooper TB Sun K Zimerman B Frazier J Williams M. Double-blind and placebo-controlled study of lithium for adolescent bipolar disorders with secondary substance dependency. J Am Acad Child Adolesc Psychiatry. (1998) 37:171–8. 10.1097/00004583-199802000-00009 9473913

[109] Fawcett J Kravitz HM McGuire M Easton M Ross J Pisani V. Pharmacological treatments for alcoholism: revisiting lithium and considering buspirone. Alcohol Clin Exp Res. (2000) 24:666–74. 10832908

[110] Brady KT Sonne SC Anton R Ballenger JC. Valproate in the treatment of acute bipolar affective episodes complicated by substance abuse: a pilot study. J Clin. Psychiatry. (1995) 56:118–21. 7883730

[111] Rubio G Lopez-Munoz F Alamo C. Effects of lamotrigine in patients with bipolar disorder and alcohol dependence. Bipolar Disord. (2006) 8:289–93. 10.1111/j.1399-5618.2006.00292.x 16696832

[112] Coles AS Sasiadek J George TP. Pharmacotherapies for co-occurring substance use and bipolar disorders: a systematic review. Bipolar Disord. (2019) 21:595–610. 10.1111/bdi.12794 31077521

[113] Brown ES Sunderajan P Hu LT Sowell SM Carmody TJ. A randomized, double-blind, placebo-controlled, trial of lamotrigine therapy in bipolar disorder, depressed or mixed phase and cocaine dependence. Neuropsychopharmacology. (2012) 37:2347–54. 10.1038/npp.2012.90 22669171 PMC3442350

[114] Sylvia LG Gold AK Stange JP Peckham AD Deckersbach T Calabrese JR. A randomized, placebo-controlled proof-of-concept trial of adjunctive topiramate for alcohol use disorders in bipolar disorder. Am J Addict. (2016) 25:94–8. 10.1111/ajad.12346 26894822 PMC4791182

[115] Kishi T Sevy S Chekuri R Correll CU. Antipsychotics for primary alcohol dependence: a systematic review and meta-analysis of placebo-controlled trials. J Clin Psychiatry. (2013) 74:e642–54. 10.4088/JCP.12r08178 23945459

[116] Anton RF Kranzler H Breder C Marcus RN Carson WH Han J. A randomized, multicenter, double-blind, placebo-controlled study of the efficacy and safety of aripiprazole for the treatment of alcohol dependence. J Clin Psychopharmacol. (2008) 28:5–12. 10.1097/jcp.0b013e3181602fd4 18204334

[117] Brown ES Jeffress J Liggin JD Garza M Beard L. Switching outpatients with bipolar or schizoaffective disorders and substance abuse from their current antipsychotic to aripiprazole. J Clin Psychiatry. (2005) 66:756–60. 10.4088/jcp.v66n0613 15960570

[118] Longoria J Brown ES Perantie DC Bobadilla L Nejtek VA. Quetiapine for alcohol use and craving in bipolar disorder. J Clin Psychopharmacol. (2004) 24:101–2. 10.1097/01.jcp.0000106230.36344.b1 14709960

[119] Brown ES Garza M Carmody TJ. A randomized, double-blind, placebo-controlled add-on trial of quetiapine in outpatients with bipolar disorder and alcohol use disorders. J Clin Psychiatry. (2008) 69:701–5. 10.4088/jcp.v69n0502 18312058

[120] Stedman M Pettinati HM Brown ES Kotz M Calabrese JR Raines S. A double-blind, placebo-controlled study with quetiapine as adjunct therapy with lithium or divalproex in bipolar I patients with coexisting alcohol dependence. Alcohol Clin Exp Res. (2010) 34:1822–31. 10.1111/j.1530-0277.2010.01270.x 20626727

[121] Brown ES Carmody TJ Schmitz JM Caetano R Adinoff B Swann AC. A randomized, double-blind, placebo-controlled pilot study of naltrexone in outpatients with bipolar disorder and alcohol dependence. Alcohol Clin Exp Res. (2009) 33:1863–9. 10.1111/j.1530-0277.2009.01024.x 19673746 PMC3040070

[122] Tolliver BK Desantis SM Brown DG Prisciandaro JJ Brady KT. A randomized, double-blind, placebo-controlled clinical trial of acamprosate in alcohol-dependent individuals with bipolar disorder: a preliminary report. Bipolar Disord. (2012) 14:54–63. 10.1111/j.1399-5618.2011.00973.x 22329472

[123] Brown ES Davila D Nakamura A Carmody TJ Rush AJ Lo A. A randomized, double-blind, placebo-controlled trial of quetiapine in patients with bipolar disorder, mixed or depressed phase, and alcohol dependence. Alcohol Clin Exp Res. (2014) 38:2113–8. 10.1111/acer.12445 24976394 PMC4107121

[124] Brown ES McArdle M Palka J Bice C Ivleva E Nakamura A. A randomized, double-blind, placebo-controlled proof-of-concept study of ondansetron for bipolar and related disorders and alcohol use disorder. Eur Neuropsychopharmacol. (2021) 43:92–101. 10.1016/j.euroneuro.2020.12.006 33402258

[125] Brown ES Beard L Dobbs L Rush AJ. Naltrexone in patients with bipolar disorder and alcohol dependence. Depress Anxiety. (2006) 23:492–5. 10.1002/da.20213 16841344

[126] Johnson BA Roache JD Javors MA DiClemente CC Cloninger CR Prihoda TJ. Ondansetron for reduction of drinking among biologically predisposed alcoholic patients: a randomized controlled trial. JAMA. (2000) 284:963–71. 10.1001/jama.284.8.963 10944641

[127] Hamilton M. A rating scale for depression. J Neurol Neurosurg Psychiatry. (1960) 23:56–62. 14399272 10.1136/jnnp.23.1.56 PMC495331

